# Vitamin K2 in multiple sclerosis patients

**DOI:** 10.1007/s00508-018-1328-x

**Published:** 2018-03-02

**Authors:** Reza Lasemi, Michael Kundi, Nahid Beladi Moghadam, Hanns Moshammer, Johannes A. Hainfellner

**Affiliations:** 1 Department for Environmental Health, Center for Public Health, Medical University of Vienna, Kinderspitalgasse 15, 1090 Vienna, Austria; 2 Neurology Department, Shahid Beheshti University of Medical Science, Tehran, Iran; 3 Brain Mapping Research Center, Shahid Beheshti University of Medical Science, Tehran, Iran; 4 Institute of Neurology, Medical University of Vienna, Vienna, Austria

**Keywords:** Menaquinones, Gender, Age

## Abstract

**Background:**

Vitamin K2 (VK2) belongs to the vitamin K family and comprises a number of subtypes differing in length of side chains consisting of isoprenoid groups (menaquinone-*n*, MK-*n*). It is essential for a number of physiological functions although the full spectrum of activity has not yet been elucidated. Due to its role in protection of mitochondrial damage, VK2 could be relevant in preventing disease progress in multiple sclerosis (MS).

**Methods:**

We measured VK2 serum levels by the double antibody sandwich Enzyme-linked Immunosorbent Assay (ELISA) technique in MS patients and age and sex matched controls, both under vitamin D supplementation, and related it to disease characteristics and treatment.

**Results:**

Overall, 45 MS patients (31 females and 39 of the relapsing-remitting type) and 29 healthy controls (19 females) were included in the analysis. The MS patients had vastly lower VK2 blood levels than controls (235 ± 100 ng/ml vs. 812 ± 154 ng/ml, respectively). Female patients had significantly lower VK2 levels than males and a decrease with age by approximately 10% per decade was found. The VK2 levels were lower with increasing numbers of attacks per year and were higher in patients with optic nerve lesions. No consistent relationship with medications was detected.

**Conclusion:**

The substantially lower levels of VK2 in MS patients could be due to depletion, lower production in the gut, diminished absorption or, less likely, reduced intake of precursor vitamin K1. The role of VK2 in MS development and progress deserves further study.

**Electronic supplementary material:**

The online version of this article (10.1007/s00508-018-1328-x) contains supplementary material, which is available to authorized users.

## Introduction

Multiple sclerosis (MS) is a chronic demyelinating disease and one of the most common disabling neurological disorders in young and middle-aged adults [1]. It has higher prevalence in women than men and is higher in the northern hemisphere. The main pathogenesis of MS is assumed to be an immune-mediated disorder of the central nervous system (CNS; [2]). Relapsing-remitting MS (RRMS) is the most frequent type comprising approximately 85% of MS patients, primary progressive MS (PPMS) with approximately 10% and progressive-relapsing MS (PRMS) with 5% are rare [1–3].

The pathophysiology of MS involves inflammatory processes of myelin sheaths of nerve cells in the CNS by compound immune attacks on oligodendrocytes causing demyelination with subsequent remyelination sustained mainly by oligodendrocyte progenitors cells (OPC; [1]). Impairment of this protecting shielding disrupts the signal pathways, causing a variety of symptoms [1]. The development of focal inflammatory lesions in the CNS is followed by axonal damage and gliosis [2].

Demyelination is due to immune cells entering the central nervous system and destroying the myelin sheath [4, 5]. Remyelination by fresh myelin sheaths produced around axons in the CNS can restore neurological function. Immigration of OPCs into the lesion and their differentiation into myelinating cells generates functional myelin [6]; however, studies have indicated that, unlike OPCs, oligodendrocytes have no role in remyelination [7]. This process may be impaired by inflammation caused by T cells producing inflammatory cytokines, such as interleukin 1 (IL1), IL6 and IL23 [8]. Prevention of these inflammatory factors or their consequences on the tissue can inhibit inflammation [9]. There are some modulators influencing these processes and vitamin K2 (VK2) can be considered an anti-inflammatory factor. Symptom onset is generally before the age of 55 years, the most prevalent age at onset lying between 20 and 40 years. Women are in general affected more frequently with an average prevalence nearly double that of men [2]. It is thought that environmental factors or the interplay between environmental factors and susceptibility genes play a decisive role in the etiology of the disease. Vitamin D status seems to be an important factor related to the environment (UV radiation and nutrition) for MS incidence, severity and progression [10, 11]. Vitamin K is predominantly known for its role in hemostasis [12] and one of the newly discovered vitamins is vitamin K2 (VK2, menaquinone) which is described as a protective factor for bones, the circulatory system and endothelium. Other roles of this vitamin on other tissues such as the nervous system have been described [13]. There are several factors that can affect development and progression of MS in which VK2 may play a protective role due to its anti-autoimmune action [14].

Vitamin K is the name for a group of fat-soluble molecules that have a common 2‑methyl-1,4-naphthoquinone nucleus and differ in the structures of the side chain at the 3‑position. They are synthesized by plants and bacteria. In plants the important molecular type is phylloquinone (vitamin K1). Bacteria synthesize compounds called menaquinones (MK, VK2), which have side chains of 1–14 repeated unsaturated isopentenyl units, with MK-*n* specifying the number of these units (*n*; [15, 16]). Menadione or K3 can be viewed as a provitamin, as vertebrates can change it to VK2 (MK-4) by adding a 4-prenyl side chain at the 3‑position [16].

Vitamin Ks are fat soluble and include vitamin K1 (phylloquinone), K2 (menaquinone) and K3 (menadione; [17]). Menaquinone comprises MK1–14 but mostly includes MK4–14 [18]. It has an effect on hemostasis, coagulation and bone metabolism, and has a protective action against oxidative injury of oligodendrocyte precursors and undeveloped neurons [19]. The main MK-*n* in the brain is MK4 and was found in significantly higher concentrations in myelinated parts (pons, medulla, midbrain) than in non-myelinated areas in rats [20]. Vitamin K in brain contributes to sphingolipid synthesis, which is involved in cell proliferation, differentiation and cell-to-cell interactions. Deterioration of sphingolipid metabolism causes cognitive decline and neurodegenerative diseases, such as Alzheimer’s disease [21]. Furthermore, development of mitochondrial dysfunction was reduced by vitamin K2, which can serve as an electron carrier helping to maintain normal adenosine triphosphate (ATP) production in the mitochondria [22, 23].

The large intestines absorb 10–20% of the total vitamin K intake, which contains vitamin VK1 (green leaves) and VK2 (meat and some plants; [24]). After absorption, vitamins VK1 and VK2 (MK-*n*) are converted to VK3 as an intermediate then tissues trap K3 and convert it to MK-4 [25]. Part of the vitamin K1 supply is converted to VK3 and VK2, mostly to MK-7 and to MK-11 by bacteria in the intestines [14, 16]. In all organs VK2 is converted to MK-4 except in the liver, which accumulates MK-*n* unchanged [14, 26–28]. Vitamin K1 and K2 (MK-4) block 12-lypoxigenase (12-LOX) activation and inhibit accumulation of reactive oxygen species (ROS). Vitamin K does not directly inhibit 12-LOX enzyme activity when evaluated with purified 12-LOX in vitro, suggesting that vitamin K prevents oxidative cell death indirectly by blocking activation of 12-LOX and ROS generation [14, 29]. Intake of VK2 (MK-4) has been shown to ameliorate experimental autoimmune encephalomyelitis in an animal model of multiple sclerosis [30]. The VK2 is considered a candidate for clinical application in patients with MS to reduce the severity of relapses or to prevent progression [30]. Because the discharge of arachidonic acid and resulting oxidative stress are related to ischemic injury and numerous neurological diseases, the practical efficacy of the natural forms of VK2 in preventing oxidative injury and to affect the clinical progress of MS could be of relevance [29].

In 2011 Crivello et al. demonstrated the presence of VK2 (MK-4) in myelin fractions and a positive correlation between MK-4 and sulfatides in brain myelin [31], suggesting the physiological role of MK-4 as a cofactor for gamma-carboxylation reactions (conversion of peptide-bound glutamic acid to gamma-carboxyglutamic acid; [32]); however, more research is needed to clarify the function of VK2 in tissues and cells. There is sufficient evidence of beneficial VK2 effects on the nervous system to encourage clinical and experimental studies on the role of VK2 in the clinical course of MS [16]. Although vitamin K participation in the brain pathology has not been fully explained, it is well known that oxidative stress has a critical role in neurodegenerative diseases [29]. Vitamin K2 seems to protect neurons and oligodendrocytes from oxidative injury and in drosophila it was shown to protect against mitochondrial damage, a pathology associated with Parkinson’s disease [33]. The MK-4 or MK-7 can suppress IL6 and MK-4 can suppress prostaglandin E2 by inhibition of cox2; therefore, these compounds can reduce inflammation and the consequences of autoimmune diseases [14].

Because of the potential role of VK2 in MS and the possibility that it may be associated with its clinical features, for the first time we assessed VK2 serum levels in MS patients in comparison to healthy controls and correlated these levels with clinical appearance, medication, and disability status.

## Material and methods

### Study design

A cross-sectional study was performed in the area of Tehran, Iran. Inclusion criteria for patients were established diagnosis of MS, no other acute or chronic disease at time of blood withdrawal determined by routine tests, such as blood chemistry measuring serum glutamic oxaloacetic transaminase (SGOT), serum glutamate-pyruvate transaminase (SGPT) and creatinine and differential blood counts and for controls inclusion criteria were no known or suspected chronic disease and no acute disease at the time of blood withdrawal. The MS patients presenting at routine check-ups were eligible if they experienced no attacks and no change of medication during the preceding month and were not currently under anti-inflammatory medication. Furthermore, controls must have been on vitamin D supplementation for at least 6 months to account for the routine supplementation provided for MS patients. Exclusion criteria for both patients and controls were previous history of vitamin K and VK2 supplementation and restricted diets. Patients were selected by simple randomization from patients scheduled for routine check-up tests (46 cases) and controls (32 samples) by simple random selection from persons scheduled for blood sampling for occupational check-ups.

Written informed consent was obtained from all participants. Ethical approval has been granted by the Brain Mapping Research Center, Shahid Beheshti University of Medical Science, Tehran, Iran (number 0460/36).

### Study participants

Overall, 45 MS (31 females and 14 males) patients with 39 RRMS, 5 secondary progressive MS (SPMS) and 1 PPMS patient and 29 healthy controls (19 females and 10 males) were included in the analyses. Of the patients one was omitted because of concomitant inflammatory diseases and three controls were omitted due to a disease detected during examination (one osteoporosis, and two others had hypothyroidism).

### Blood sampling

Blood sampling was carried out in patients who needed routine laboratory tests at scheduled visits so that no additional blood withdrawals were necessary for study purposes. Samples were stored in the Massoud laboratory (Tehran) at −20 °C until analysis 2–4 weeks later. In controls, blood sampling was performed in the same laboratory stored and analyzed under the same conditions as for MS patients.

### Measurement of vitamin K2

The technique used involved 2×48-well Enzyme-linked Immunosorbent Assay (ELISA) VK2 human kits based on the biotin double antibody sandwich technology (Biotrend, Cologne, Germany). The assay measures all varieties of VK2 (MK-*n*) within a range of 5–1000 ng/ml. The VK2 kit includes a standard solution from which concentrations of 40, 80, 160, 320, 640 ng/ml were prepared with serial dilutions for the standard curve. A 4-parameter logistic function was fitted for interpolation after assessment by the ELISA reader (at 450 nm); however, in healthy controls the upper limit of 1000 ng/ml was often exceeded. Therefore, an inverse variance weighted extrapolation was performed. The intra-assay coefficient of variance (CV) is below 3%, the inter-assay CV is below 7%.

### Questionnaire

In patients and controls a short questionnaire assessing sociodemographic data was applied. In addition, a clinical questionnaire applied in MS patients assessed duration since first diagnosis, number and type of attacks, location and number of lesions, medication and sun exposure. In addition, the Expanded Disability Status Scale (EDDS; [34]) was used in MS patients.

### Statistical analysis

Sample size was determined by assuming an average difference between MS patients and controls of one standard deviation, a two-sided significance level of 5% and a power of 90%. Under these conditions 23 patients per group are required; however, because of the intention to also test the impact of clinical features by multiple regression analysis, a sample size of *n* = 45 of the MS group is sufficient to detect a relationship associated with a partial R^2^ = 0.36 (corresponding to a partial correlation coefficient of 0.6) at the 5% level of significance with a power of 90%.

The VK2 concentrations were log transformed and analyzed by the general linear model (GLM) with group (MS patients vs. controls) and gender as factors and age as a control variable. Furthermore, within MS patients GLM regression analyses were performed with demographic and clinical parameters as predictors of VK2 concentration and others assessing attack characteristics and medication. All analyses were performed by SPSS 23.0 (IBM, New York, NY, USA) and *p*-values below 0.05 were considered significant.

## Results

The MS patients and controls did not differ with respect to age and gender distribution. The age of controls was 34 years on average (median, interquartile range, IQR = 26–42 years) and was the same in MS patients (median 34 years, IQR = 30–41 years). Approximately two thirds (66% and 69%, respectively) of participating controls and MS patients were females. Sun exposure in both groups was low and amounted to less than a 15 min per day in both groups (Table 1).

**Table 1:** Characteristics of MS patients and controls (median and interquartile range or percentage)

	Controls (*n* = 29)	MS patients (*n* = 45)	*p* value
Age (years)	34 (26–42)	34 (30–41)	0.825
Gender (females)	66%	69%	0.762
Sun exposure (min/day)	13 (10–18)	10 (10–15)	0.479

The majority of MS patients (27, 60%) had lesions in more than one region. Spinal lesions were present in 35 patients (78%), lesions in the optic tract in 22 (49%), in the cerebellum in 15 (33%) and in the brain stem in 6 (13%) patients. No difference between genders was noted with respect to lesion locations. Disease duration ranged between 1 and 25 years (median 6 years). During the last year patients had experienced 0–5 attacks (median 2), most often fatigue (*n* = 38, 84%) and blurred vision (*n* = 9, 20%), with a duration between 0.5 and 5 weeks (median 2 weeks; Supplementary table 1).

The VK2 serum levels were more than three-fold higher in healthy controls as compared to MS patients (*p* < 0.001): healthy controls had a median level of 866 ng/ml (IQR = 780–901 ng/ml) and a geometric mean (95% confidence interval, CI) of 785 ng/ml (713–865 ng/ml), compared to a median level of 196 ng/ml (IQR = 178–238) and a geometric mean of 229 ng/ml (212–248 ng/ml) in MS cases. By application of a GLM with age and gender as control variables, an interaction with gender was detected (Table 2) with higher levels in control females but lower levels in MS females. In addition, age had an effect with a 10% decline in VK2 levels by a decade increase of age that was more pronounced in MS patients (Table 2).

**Table 2:** Vitamin K2 serum levels (mean and 95% confidence intervals) by group (controls vs. MS patients), gender and age, results of the General Linear Model (Wald’s χ^2^-test, *p*-value)

Source of variation		Vitamin K2 (ng/ml)	Wald’s χ^2^-test	*p* value
Controls vs. MS (group)	Controls	785 (713–865)	359.55	<0.001
	MS patients	229 (212–248)		
Gender	Males	432 (389–479)	0.55	0.460
	Females	417 (388–448)		
Group * gender	Controls: males	723 (616–848)	7.56	0.006
	Controls: females	852 (761–954)		
	MS: males	258 (226–294)		
	MS: females	204 (187–223)		
*Change of VK2 levels (%)*				
Age	By 10 years of age	−9.8% (−15.5 to −3.7%)	5.71	0.017
Group * Age	Controls by 10 years of age	−1.5% (−7.6 to +9.8%)	3.82	0.051
	MS by 10 years of age	−15.1% (−24.1 to −4.9%)		

**Fig. 1:**
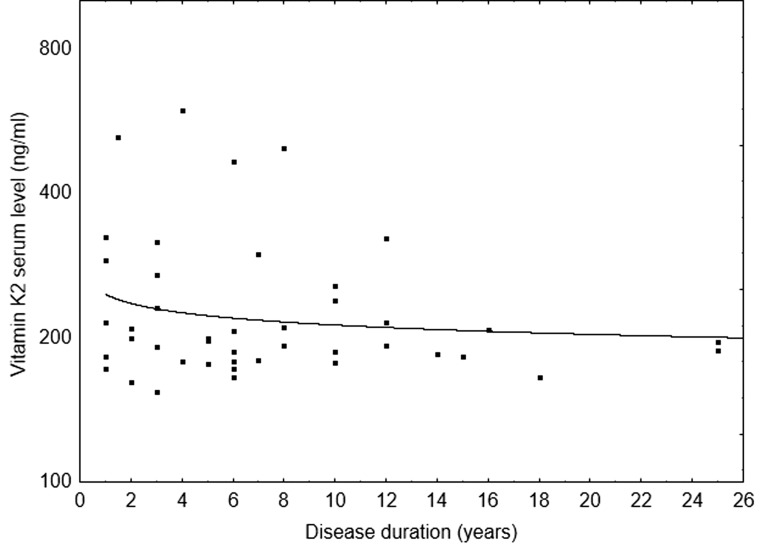
Vitamin K2 serum levels in multiple sclerosis patients as a function of disease duration

While multivariate analysis did not indicate a relationship between VK2 serum levels and duration of the disease, this was due to the high correlation between age and disease duration. In fact, VK2 serum levels declined with duration since diagnosis of MS (Fig. 1) that could, however, not be discriminated from the effect of age.

Patients showed no dependency of VK2 serum levels on type of disease (RR, PP or SP); however, SP and PP were rare. Increasing numbers of attacks per year were associated with reduced VK2 serum levels, an increase of one attack per year reduced the VK2 serum level by 10%. Higher sun exposure tended to increase VK2 serum levels (Table 3). Although higher disability as assessed by the EDSS was associated with somewhat lower VK2 levels this relationship was not statistically significant in the multivariate analysis (*p* = 0.208) due to collinearity with age (r = 0.6) and disease duration (r = 0.7).

**Table 3:** Impact of disease features of MS patients and demographic characteristics on vitamin K2 serum levels. % change and 95% confidence intervals (CI) by group or increase by one unit. Parameter estimates and *p*-values from the general linear model

Parameter	% Change (95% CI)	Coefficient	SE	*p* value
Relapsing-remitting vs. secondary progressive	+13 (−19 to +58)	0.053	0.074	0.474
Relapsing-remitting vs. primary progressive	−4 (−52 to +93)	−0.019	0.155	0.901
Gender (males vs. females)	+29 (+9 to +53)	0.111	0.038	0.004
Age (decade)	−15 (−24 to −5)	−0.071	0.025	0.005
Sun exposure (min/day)	+1 (0 to +3)	0.005	0.003	0.099
Duration of disease (years)	+1 (−1 to +4)	0.006	0.005	0.271
Attacks/year	−10 (−19 to −1)	−0.046	0.023	0.043
Lesion regions (*n*)	+14 (−2 to +31)	0.055	0.032	0.086
Expanded Disability Status Score	−10 (−25 to +7)	−0.048	0.039	0.208

*CI* confidence interval, *SE* standard error

A similar analysis of additional clinical features revealed that visual type attacks (blurred vision) are associated with reduced VK2 values but since patients with a lesion in the optic tract had significantly higher VK2 values, this may reflect the impact of more frequent attacks (Table 4).

**Table 4:** Impact of attack characteristics on vitamin K2 (VK2) serum levels (mean and 95% confidence interval). Parameter estimates and *p*-values from the general linear model

Variable	Category	*n*	VK2 (ng/ml)	Coefficient	SE	*p* value
Location of lesion	Spinal	35	199 (147–269)	−0.007	0.049	0.883
	Brain stem	6	197 (143–270)	0.005	0.052	0.923
	Cerebellum	15	199 (155–254)	−0.014	0.041	0.738
	Optic nerve	22	230 (177–298)	0.126	0.036	0.001
Type of attack	Fatigue	38	194 (157–240)	−0.044	0.051	0.388
	Impaired vision	9	186 (137–253)	−0.099	0.048	0.041
	Ataxia	6	202 (146–280)	0.022	0.057	0.701
	Paresthesia	4	191 (137–268)	−0.069	0.056	0.216
Attack duration	% Change by week	–	−6% (−14 to +3%)	−0.030	0.019	0.111

*SE* standard error

## Discussion

The normal range of VK2 has not yet been established, therefore, healthy age and gender matched controls were included in this study. Highly significant differences in VK2 serum concentrations between controls and MS patients were found (*p* < 0.001) with controls showing more than three-fold higher levels on average. To our knowledge, this is the first study evaluating VK2 levels in MS patients and therefore these pronounced differences cannot be attributed to any established factor. They could be due to increased consumption by tissues for VK2 anti-inflammatory and anti-demyelinating activity [29] or to decreased production of vitamin K2 and vitamin K1 by changes in intestinal flora [35] or to diminished absorption in the intestines [36]. Any or all of these mechanisms may be involved and further research is needed to clarify this issue. This will be of great importance given the potential impact on the course of the disease. The significant relationship with the number of attacks points to a progressive impediment of VK2 tissue availability.

There was a significant difference between genders in VK2 serum levels of MS patients with males having higher VK2 values than females, while in healthy controls the opposite was the case. This difference might be due to the 2 years longer disease duration on average in females. Age was also related to VK2 serum levels with increasing age associated with decreasing serum levels. These differences may be due to differences in nutritional habits and differences in intestinal flora and absorption; however, it has to be noted that the decline by age was significant in MS patients only, with an annual decline rate of about 15%. Although there was no significant effect of disease duration, there was a clear declining trend with increasing disease duration that could, however, not be distinguished from the effect of age due to the strong correlation between age and duration of the disease. Since no decline of VK2 serum levels with increasing age could be established in controls, the age trend in MS patients can be interpreted as reflecting the impact of prolonged duration of the disease.

We found no differences in VK2 serum concentrations according to type of disease (RR, PP or SP); however, only 6 patients were not of the RR type and, hence, no conclusions can be drawn from this result.

Visual problems are the most prevalent symptoms among MS patients [37]. We found blurred vision to be associated with lower levels of VK2. It is noteworthy that none of these patients had oxybutynin as medication since blurred vision could be a side effect of this drug [38]. On the other hand, patients with a lesion in the optic tract had significantly higher VK2 values. The diverse patterns of medications prohibit any firm conclusion about the impact of medication on VK2 levels; however, we found no consistent relationship between medication and VK2 levels when clinical features were taken into account.

## Conclusion

Information about the activity of vitamin K2 is new and developing but it has been implicated as an anti-demyelinating and anti-inflammatory agent. We have for the first time demonstrated a pronounced lower concentration of VK2 in serum of MS patients. This can be due to increased consumption of VK2 by the damaged tissue or to changes in intestinal production and absorption. Nutritional differences, although unlikely, cannot be ruled out as well. Clarification of the reasons for this difference is of great importance because use of VK2 as a means to inhibit disease progress will depend on the process leading to reduced serum levels. Reduced levels with increasing number of attacks and longer disease duration point to a depletion of VK2 in the affected tissue. Discrimination between the different mechanisms may be possible by measuring the VK2 level in liquor and its relation to serum levels.

## Caption Electronic Supplementary Material

Supplementary Table 1. Patients characteristics
