# Supplementary material for: Vitamin K2 in multiple sclerosis patients
Source: Wien Klin Wochenschr. 2018 Mar 2;130(9):307–13. doi: 10.1007/s00508-018-1328-x (PMC5966473; doi:10.1007/s00508-018-1328-x)
Supplement: Supplementary file 1 — Supplementary Table 1. Patients characteristics [file 508_2018_1328_MOESM1_ESM.docx]

Supplementary Table 1. Patients characteristics

| Nr | Gender | Age (years) | MS Type | Duration (years) | Location of lesions | # Attacks  last year | # Lesion locations | Attack type | Attack duration (weeks) | Sun exposure (min/d) | Medication during last year |
| --- | --- | --- | --- | --- | --- | --- | --- | --- | --- | --- | --- |
| 1 | Male | 25 | RR | 2 | Spinal | 1 | 1 | Fatigue | 3 | 10 | IFN ß 1a |
| 2 | Male | 50 | RR | 10 | Optic tract | 1 | 1 | Visual | 2 | 25 | glatiramer |
| 3 | Female | 61 | PP | 25 | Spinal/Cerebellum | 2 | 2 | Fatigue | 2 | 32 |  |
| 4 | Female | 56 | SP | 16 | Spinal/Optic tract | 2 | 2 | Fatigue | 3 | 5 | gabapentin, bethanechol |
| 5 | Female | 23 | RR | 12 | Spinal/Cerebellum/Optic tract | 2 | 3 | Fatigue | 2 | 10 | IFN ß 1b, amantadine |
| 6 | Female | 18 | RR | 3 | Optic tract | 3 | 1 | Fatigue/Visual | 2 | 15 | IFN ß 1b, prednisone |
| 7 | Female | 34 | RR | 10 | Spinal/Cerebellum/Optic tract | 3 | 3 | Fatigue | 2 | 25 | fingolimod |
| 8 | Male | 50 | RR | 10 | Spinal/Cerebellum/Optic tract | 2 | 3 | Fatigue/Visual | 3 | 20 | fingolimod |
| 9 | Female | 20 | RR | 6 | Spinal/Optic tract | 1 | 2 | Paresthesia | 1 | 15 | IFN ß 1a |
| 10 | Male | 34 | RR | 2 | Spinal | 1 | 1 | Fatigue/Paresthesia | 2 | 10 | IFN ß 1b, prednisone |
| 11 | Female | 33 | RR | 3 | Cerebellum/Optic tract | 2 | 2 | Ataxia | 3 | 15 | IFN ß 1a, gabapentin, oxybutynin |
| 12 | Male | 27 | RR | 4 | Spinal/Optic tract | 0 | 2 |  |  | 15 | IFN ß 1a |
| 13 | Female | 40 | RR | 6 | Spinal/Optic tract | 1 | 2 | Fatigue | 1 | 5 | fingolimod, gabapentin |
| 14 | Female | 25 | RR | 3 | Spinal | 2 | 1 | Fatigue | 2 | 10 | IFN ß 1a, gabapentin |
| 15 | Male | 33 | RR | 1.5 | Spinal/Optic tract | 1 | 2 | Fatigue | 0.5 | 15 | IFN ß 1a |
| 16 | Female | 44 | RR | 10 | Spinal/Cerebellum/Optic tract | 3 | 3 | Fatigue/Ataxia | 2 | 10 | fingolimod, amantadine, oxybutynin |
| 17 | Female | 37 | SP | 8 | Spinal/Cerebellum | 3 | 2 | Fatigue | 1 | 5 | azathioprine, gabapentin, baclofen, oxybutynin |
| 18 | Male | 36 | RR | 3 | Optic tract | 5 | 1 | Fatigue/Visual | 3 | 5 | IFN ß 1a, oxybutynin |
| 19 | Female | 29 | RR | 6 | Optic tract | 1 | 1 | Fatigue | 1 | 10 | glatiramer, amantadine, oxybutynin |
| 20 | Female | 29 | RR | 1 | Spinal/Optic tract | 1 | 2 | Fatigue | 2 | 10 | IFN ß 1a |
| 21 | Female | 46 | RR | 12 | Spinal/Cerebellum/Optic tract | 2 | 3 | Fatigue/Ataxia | 3 | 15 | IFN ß 1a, glibenclamide, sertraline, amantadine |
| 22 | Female | 33 | RR | 5 | Spinal/Cerebellum | 2 | 2 | Fatigue | 1 | 20 | glatiramer, amantadine |
| 23 | Female | 40 | SP | 12 | Spinal | 3 | 1 | Fatigue/Paresthesia | 1 | 20 | IFN ß 1b, gabapentin, oxybutynin |
| 24 | Female | 31 | RR | 5 | Spinal/Cerebellum | 2 | 2 | Vertigo | 2 | 10 | IFN ß 1a |
| 25 | Female | 34 | RR | 5 | Spinal | 2 | 1 | Fatigue | 2 | 18 |  |
| 26 | Female | 37 | RR | 6 | Brain stem/Cerebellum | 3 | 2 | Fatigue/Paresthesia | 3 | 12 | fingolimod, oxybutynin |
| 27 | Female | 31 | RR | 1 | Spinal | 2 | 1 | Fatigue/Visual | 1 | 20 | glatiramer |
| 28 | Female | 41 | RR | 6 | Brain stem/Cerebellum | 2 | 2 | Fatigue/Ataxia | 2 | 5 | IFN ß 1b, gabapentin, sertraline, amantadine |
| 29 | Female | 33 | RR | 4 | Spinal | 2 | 1 | Fatigue | 2 | 15 | IFN ß 1a, oxybutynin |
| 30 | Female | 32 | RR | 2 | Spinal/Brain stem | 2 | 2 | Fatigue | 1 | 10 | IFN ß 1a, trazodone, sertraline, oxybutynin |
| 31 | Female | 17 | RR | 1 | Optic tract | 1 | 1 | Visual | 2 | 10 | IFN ß 1a, amantadine |
| 32 | Female | 31 | RR | 3 | Spinal/Brain stem | 2 | 2 | Fatigue | 2 | 15 | glatiramer, tizanidine, bethanechol |
| 33 | Male | 26 | RR | 8 | Brain stem/Optic tract | 1 | 2 | Fatigue | 0.5 | 10 | IFN ß 1a, cinnarizine |
| 34 | Male | 34 | RR | 1 | Spinal | 1 | 1 | Fatigue | 1 | 5 | IFN ß 1a |
| 35 | Female | 30 | RR | 7 | Spinal/Brain stem | 1 | 2 | Fatigue | 1 | 10 |  |
| 36 | Female | 32 | RR | 6 | Optic tract | 1 | 1 | Visual | 2 | 20 | IFN ß 1b, clonazepam |
| 37 | Male | 52 | RR | 7 | Spinal | 2 | 1 | Fatigue | 2 | 25 | IFN ß 1a |
| 38 | Female | 62 | SP | 25 | Spinal/Cerebellum | 3 | 2 | Fatigue/Ataxia | 4 | 10 | prednisone, gabapentin, baclofen, amantadine |
| 39 | Female | 47 | RR | 10 | Spinal/Optic tract | 2 | 2 | Fatigue | 3 | 5 | fingolimod, tizanidine |
| 40 | Male | 56 | RR | 18 | Spinal | 2 | 1 | Fatigue/Paresthesia | 1 | 10 | IFN ß 1b, gabapentin |
| 41 | Male | 40 | SP | 14 | Spinal/Cerebellum | 2 | 2 | Fatigue/Ataxia | 2 | 5 | IFN ß 1b, amantadine, bethanechol |
| 42 | Male | 37 | RR | 5 | Spinal/Cerebellum/Optic tract | 2 | 3 | Fatigue/Visual | 5 | 15 | fingolimod, oxybutynin |
| 43 | Female | 56 | RR | 15 | Spinal | 1 | 1 | Fatigue | 2 | 10 | IFN ß 1b, baclofen |
| 44 | Female | 31 | RR | 8 | Spinal/Optic tract | 2 | 2 | Fatigue/Visual | 2 | 5 | IFN ß 1a, glatiramer |
| 45 | Male | 18 | RR | 1 | Spinal | 2 | 1 | Fatigue | 3 | 10 | IFN ß 1a, amantadine |

RR…Relapsing-remitting type; PP…Primary-progressive type; SP…Secondary-progressive type
